# Near-infrared spectroscopy to assess tissue oxygenation in patients with polytrauma: relationship with outcome

**DOI:** 10.1186/cc14388

**Published:** 2015-03-16

**Authors:** A Donati, E Damiani, R Domizi, S Pierantozzi, S Calcinaro, P Pelaia

**Affiliations:** 1 Università Politecnica delle Marche, Ancona, Italy

## Introduction

We evaluated tissue oxygenation by means of near-infrared spectroscopy (NIRS) and explored its relationship with outcome in polytrauma patients.

## Methods

A prospective observational study; 37 polytrauma patients underwent NIRS monitoring (thenar eminence) every day during their stay in the ICU. A VOT was performed with a 40% tissue oxygen saturation (StO_2_) target. Healthy volunteers (*n *= 27) were studied as controls.

## Results

**Figure 1:**
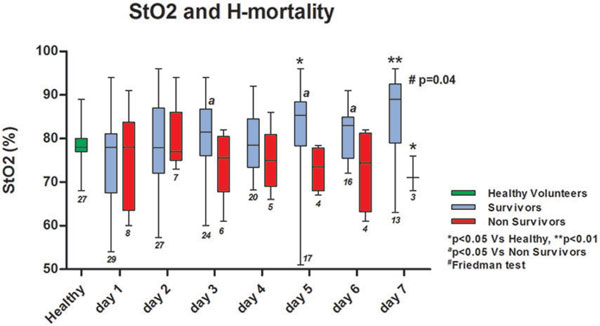


StO_2_ increased over the first 7 days only in hospital survivors (*n *= 29), who showed higher values as compared with healthy volunteers at days 5 and 7 (Figure 1). StO_2_ downslope and upslope tended to be lower in H-nonsurvivors (*n *= 8) (*P *< 0.05 at days 2 and 4) as compared with H-survivors. Tissue hemoglobin index was lower in H-no survivors over the first 7 days and tended to normalize only in H-survivors (*P >*0.05 vs. healthy at day 7). Five patients were discharged from the ICU but did not survive until H-discharge. At discharge from the ICU, these patients were similar to H-survivors in SOFA score, heart rate, mean arterial pressure and lactate, but showed lower StO_2_ downslope (-13 (-16.5, -11.7)%/minute vs. -8.6 (-11.7, -6.5)%/minute, *P *= 0.01).

## Conclusion

An increase in StO_2_ and lower tissue oxygen extraction rates were associated with H-survival in polytrauma patients.

